# Molecular mechanism of acetylsalicylic acid in improving learning and memory impairment in APP/PS1 transgenic mice by inhibiting the abnormal cell cycle re-entry of neurons

**DOI:** 10.3389/fnmol.2022.1006216

**Published:** 2022-10-03

**Authors:** Pei-Pei Guan, Wei-Yan Ding, Pu Wang

**Affiliations:** College of Life and Health Sciences, Northeastern University, Shenyang, China

**Keywords:** cell cycle re-entry, acetylsalicylic acid, apoptosis, lysosomal biogenesis, autophagy

## Abstract

Alzheimer’s disease (AD) is a neurodegenerative disorder accompanied by the loss and apoptosis of neurons. Neurons abnormally enter the cell cycle, which results in neuronal apoptosis during the course of AD development and progression. However, the mechanisms underlying cell cycle re-entry have been poorly studied. Using neuroblastoma (N) 2a*^SW^* and APP/PS1 transgenic (Tg) mice as *in vitro* and *in vivo* AD models, we found that the expression of cyclin-dependent kinase (CDK)1/2/4 and cyclin A2/B1/D3/E1 was increased while the protein expression of p18 and p21 was decreased, which led to enhanced cell cycle re-entry in a β-amyloid protein (Aβ)-dependent mechanism. By preparing and treating with the temperature-sensitive chitosan-encapsulated drug delivery system (CS), the abnormal expression of CDK1/2/4, cyclin A2/B1/D3/E1 and p18/21 was partially restored by acetylsalicylic acid (ASA), which decreased the apoptosis of neurons in APP/PS1 Tg mice. Moreover, CDK4 and p21 mediated the effects of ASA on activating transcription factor (TF) EB *via* peroxisome proliferator-activated receptor (PPAR) α, thus leading to the uptake of Aβ by astrocytes in a low-density lipoprotein receptor (Ldlr)-dependent mechanism. Moreover, the mechanisms of Aβ-degrading mechanisms are activated, including the production of microtubule-associated protein light chain (LC) 3II and Lamp2 protein by ASA in a PPARα-activated TFEB-dependent manner. All these actions contribute to decreasing the production and deposition of Aβ, thus leading to improved cognitive decline in APP/PS1 Tg mice.

## Introduction

Alzheimer’s disease (AD) is a neurodegenerative disease characterized by progressive memory loss and cognitive impairment with the deposition of β-amyloid protein (Aβ) in β-amyloid plaques (APs) and hyperphosphorylated tau in neurofibrillary tangles (NFTs) (Laferla and Oddo, 2005). Progressively, the neurons show a vacuolar degeneration and neuronal loss morphology (Laferla and Oddo, 2005). Therefore, the hypothesis of cell cycle re-entry was proposed to define the association between neurodegenerative diseases and neuronal loss (Khurana and Feany, 2007). For a long time, researchers believed that most differentiated cells will remain in the G0 phase of the cell cycle, except for cells that need active division, such as bone marrow stem cells and digestive tract mucosal cells. In the mature central nervous system (NCS), neurons are generally considered to be in the terminal state of differentiation, which means that they no longer enter the cell cycle. However, an increasing number of studies have shown that the neuronal cell cycle can abnormally restart under some pathological conditions, such as neurodegenerative disease and cerebral ischemia (Liu and Greene, 2001). For example, the expression of cyclin B and cyclin D was significantly higher in patients with mild cognitive impairment (MCI) and AD than in corresponding control subjects (Yang et al., 2003). Moreover, microchromosome maintenance complex component 2 (MDM2), which is an essential protein for DNA replication, was identified in AD patients (Bonda et al., 2009), and it can be phosphorylated by CDKs and CDC7 in S phase (Bonda et al., 2009). Immunohistochemistry (IHC) analysis showed that MDM2 was located around NFTs (Bonda et al., 2009), which provides definite evidence for aberrant cell cycle re-entry in AD neurons.

Indeed, cell cycle re-entry appears to occur earlier than the formation of APs and NFTs (Evans, 2007). Until now, there have been a series of methods to induce the cell cycle re-entry of neurons, through which its relationship with AD has been investigated. Studies have shown that mature neurons are forced to enter the cell cycle by overexpressing the antigen Simian Virus 40 T, which results in the formation of APs and NFTs. In addition, most neurons reached G2 phase according to the BrdU assay after injecting c-myc and mutated ras into primary cultured cortical neurons. More importantly, tau protein is phosphorylated and undergoes conformational changes, which is similar to the pathological changes of tau protein in AD (McShea et al., 2007). Reciprocally, Aβ treatment and tau*^P^*^301*L*^ expression in an AD tissue culture model act synergistically to promote aberrant cell cycle re-entry (Hoerndli et al., 2007). After cell cycle re-entry, neurons cannot continue to differentiate but induce the signaling cascades of apoptosis based on the expression of caspase 3 (Love, 2003), thus leading to neuronal loss during the course of AD development and progression.

Based on these clues, accumulating evidence suggests that factors that induce inhibition or block the progression of the cell cycle can protect neurons from death in AD, which provides a potential strategy for the treatment of AD (Snape et al., 2009). In other words, blocking cell cycle re-entry will provide insights into AD treatment. Retinoic acid (RA) can block the progression of the cell cycle to the G0/G1 phase by upregulating the expression of p62 and p56 in neurons (Lamkin et al., 2006). Similarly, simvastatin (Murakami et al., 2001), taurine (Chen et al., 2004), interleukin (IL) (Morinaga et al., 1990), and interferon (Sangfelt et al., 1999) can block the cell cycle to the G0/G1 phase, which exerts neuroprotective effects on neurons in AD.

Apart from the above interventions, epidemiological investigations have shown that long-term administration of acetylsalicylic acid (ASA) obviously decreases the risk of AD (Pomponi et al., 2008), suggesting its potential application prospect for combating the disease. Evidence has shown that ASA has the ability to block the effects of Aβ_1–40_ on releasing IL-6 and tumour necrosis factor (TNF)-α, thus leading to improved learning and memory. Moreover, Tortosa et al. (2006) found that ASA can inhibit the phosphorylation of tau and the formation of NFTs. Asanuma et al. (2001) pointed out that ASA is capable of clearing nitric oxide (NO), through which it protects neurons from oxidative stress-induced impairment. Moreover, Legler et al. (2010) confirmed that ASA can reduce the synthesis of prostaglandin (PG) E_2_ by inhibiting platelet activation, which alleviates inflammatory injury in neurons.

In addition to these functions, ASA may be also involved in regulating the clearance of Aβ during the course of AD development and progression. During this process, transcription factor (TF) EB overexpression potentially contribute to decrease the accumulation of Aβ *via* autophagic lysosome-degrading pathways, leading to alleviation of the progression of AD (Zhang and Zhao, 2015). Notably, peroxisome proliferator-activated receptor (PPAR)α is reported to mediate the transcriptional synthesis of TFEB in brain cells (Ghosh et al., 2015), suggesting the important roles of PPARα and TFEB pathways in mediating Aβ clearance.

Although ASA may be beneficial for AD, its effects on the cell cycle re-entry of neurons are not thoroughly known, rather than its inherent mechanisms. As a water-insoluble drug, ASA shows relatively high toxicity and side effects, which restricts its practical applications. Recently, *in vivo* temperature-sensitive chitosan gel has progressively become a good drug delivery system for decreasing the toxicity and side effects of drugs (Eve and Leroux, 2004). In detail, chitosan and glycerol phosphate are used to prepare thermosensitive hydrogels loaded with adriamycin (an effective drug for treating malignant tumors), which achieve better therapeutic effects by decreasing toxicity and side effects. Under the condition of long-term administration, nasal mucosal administration has a natural advantage to treat AD by bypassing the blood–brain barrier (BBB) through the olfactory nerve and directly reaching brain tissue.

Based on the above clues, the current study aimed to reveal the regulatory mechanisms of cell cycle re-entry during the course of AD development and progression. Taking advantage of the ASA-CS drug delivery system, we revealed that ASA protects neurons from apoptosis by inhibiting cell cycle re-entry. In addition, we described the roles of ASA in disrupting the deposition of Aβ in APs and inducing the uptake of Aβ for degradation in the astrocytes of APP/PS1 transgenic (Tg) mice. All these actions mediate the effects of ASA on improving the cognitive decline of AD animals.

## Materials and methods

### Reagents

Acetylsalicylic acid, chitosan, Aβ, GW6471 and β-glycerphosphate were obtained from Sigma-Aldrich (Shanghai, China). Antibodies specific for CDK4, cyclin E1 and p21 were obtained from ImmunoWay Biotechnology Company (Suzhou, Jiangsu, China). Antibodies for low-density lipoprotein receptor (Ldlr) and PPAR α were purchased from Abcam (Shanghai, China). Antibodies for Aβ, CDK1, CDK2, cyclin A2, Cyclin B1, Cyclin D3, p18, Caspase 3, NeuN, PSD95, SYP, Bax, Bcl-2, GFAP, TFEB, LC3, Lamp2, histone, and β-actin were purchased from Cell Signaling Technology (Shanghai, China). Aβ_1–40_ and Aβ_1–42_ immunoassay kits were purchased from Invitrogen (Shanghai, China). The kits for RNA extraction, reverse transcription and real-time PCR were obtained from Promega Corporation (Beijing, China). The kits for IHC were purchased from MXB Biotechnologies (Fuzhou, Fujian, China). All other reagents were from Thermo Fisher Scientific (Shanghai, China) and Fuyu Chemical (Tianjin, China) unless specified otherwise.

### Cell culture

D1A, neuroblastoma (N) 2a, N2a*^WT^* and N2a*^SW^* cells were cultured in 5% CO_2_ at 37°C on 6 cm tissue culture dishes in dulbecco’s modified eagle medium (DMEM) culture medium. In a select set of experiments, N2a cells were incubated in serum-deprived medium for an additional 24 h before treatment with the indicated concentration of ASA (5 or 10 μM) in the absence or presence of Aβo (20 nM). In a separate set of experiments, N2a cells were transfected with CDK4 shRNA or p21 cDNA before treatment with Aβo. In a separate set of experiments, D1A cells were treated with ASA (10 μM) in the absence or presence of the PPARα antagonist GW6471 (1 μM) or transfected with shRNA targeting TFEB or p21 or cDNA constructs encoding CDK4 coding sequences. After treatment, the cells were lysed for RNA or protein extraction or stained with 3-(4,5-Dimethylthiazol-2-yl)-2,5-diphenyltetrazolium bromide (MTT) assay (3 h at 37°C).

### Animals

Wild-type (WT) mice were purchased from Liaoning Chengda Biotechnology Co., Ltd. (Benxi, Liaoning, China). APP/PS1 (Stock No. 004462) Tg mice were obtained from the Jackson Laboratory (Bar Harbor, ME, USA). Genotyping was per-formed after 1 month of birth. The mice were then randomly divided into different groups, which are, respectively treated with vehicle, ASA solution or ASA CS (0.2 mg/kg/d) for 3 months. The general health and body weights of the animals were monitored every day. The Morris maze test and nest construction were performed before collecting brains under anesthesia.

### Preparation of the thermosensitive chitosan gels

First, 200 mg of CS (medium viscosity, degree of deacetylation is 91) was dissolved in hydrochloric acid and acetic acid (v:v, 4:1) solution under vigorous stirring. After CS was dissolved, the solution was cooled down by placing it on ice for 20 min. Then, 200 mg of β-glycerphosphate (GPS) in 0.4 ml of distilled water was slowly added to the CS solution. The mixture was heated in a 37°C water bath, after which thermosensitive chitosan gels were formed in a few minutes. To prepare ASA-CS, a certain amount of ASA was added to a 2% CS solution. After dissolving by oscillation, GPS was added to the solution to mix together on ice. Then, the mixture will be placed in a 37°C water bath until ASA-CS gels form.

### Protein extraction and western blots

A total of 100 μl of lysis buffer was added to cell pellets or tissues with the inhibitors of 1 μl proteinase and 1 μl phosphatase. After smashing with 1 ml syringe, the samples were vortexed on ice every 10 min. After 1 h, the tubes were centrifuged at 15, 000 rpm for 15 min at 4°C. The supernatants were collected and stored at −80°C for use. The protein concentration was measured by BCA kits (Pierce, Shanghai, China) and calculated by standard curve. The samples were diluted according to the protein concentration, which was loaded in SDS-PAGE. After transferring to polyvinylidene fluoride (PVDF) membrane, the protein was probed with specific antibody. After developing, the specific band was visualized by ECL (Tanon, Shanghai, China).

### RNA extraction and real-time PCR

The cells and tissues were crushed by ultrasonication in 1 ml TRIzol on ice. After vortexing for 30 s, 0.2 ml of chloroform was added to the tube and then vortex vigorously for 15 s. After centrifugation at 12, 000 rpm for 10 min, the supernatant was transferred to a new tube. The RNA was then purified by RNA extraction kits (Thermo Fischer Scientific, Shanghai, China). After analyzing the concentration of RNA with NanoDrop microvolume spectrophotometer (Thermo Fischer Scientific, Shanghai, China), the RNA was diluted and used for the real-time PCR assays. In brief, real-time PCR assays were performed with the MiniOpticon Real-Time PCR detection system (Bio-Rad Laboratories, Beijing, China) with Real-Time PCR kits and the appropriate primers. The sequences of primers were listed in Table 1. The gene expression values were normalized to that of GAPDH.

**TABLE 1:** The list for the sequences of primers.

Genes	F/R	Sequences
CDK1	Forward	CCAAGAAGCCGCTTTTCCAC
	Reverse	AAAGTACGGGTGCTTCAGGG
CDK2	Forward	TACCCAGTACTGCCATCCGA
	Reverse	GACACGGTGAGAATGGCAGA
CDK4	Forward	GGAGGCCTTTGAACATCCCA
	Reverse	GTTCTCTGGCTTCAGGTCCC
CyA2	Forward	GTCAACCCCGAAAAACTGGC
	Reverse	CAGCTGGCCTCTTCTGAGTC
CyB1	Forward	TCTCCAAGCCCGATGGAAAC
	Reverse	ACATGGTCTCCTGAAGCAGC
CyD3	Forward	AAACAGATGTCCTGCAGCGA
	Reverse	TGTGCGGCTTGATCTCCTTT
CyE1	Forward	GAAAAGCGAGGATAGCAGTCAG
	Reverse	CCCAATTCAAGACGGGAAGTG
p18	Forward	GATTTGGGAGAACTGCGCTG
	Reverse	TGCAGGCTGTGTGCTTCATA
p21	Forward	GTACTTCCTCTGCCCTGCTG
	Reverse	CTGACCCACAGCAGAAGAGG
GAPDH	Forward	TTCACCACCATGGAGAAGGC
	Reverse	AGTGATGGCATGGACTGTGG

### Enzyme-linked immunosorbent assay

The mouse Aβ_1–40_ and Aβ_1–42_ kits were obtained from Thermo Fisher Scientific (Shanghai, China). The contents of Aβ_1–40_ and Aβ_1–42_ were measured according to the manufacturer’s instructions. In brief, samples, standards or controls were added into the wells, which bind to the immobilized antibody specific for Aβ_1–40_ or Aβ_1–42_. By sequentially adding the secondary antibody and substrate solution, the contents of Aβ_1–40_ and Aβ_1–42_ were calculated according to the standard curve.

### Flow cytometry

The cells were collected with 0.05% trypsin. After centrifugation at 1,000 rpm for 5 min, the cells were immobilized by 1 ml of 70% ethanol at −4°C for 2 h. Then, the cells were stained with PI in the dark before analysis using a BD Accuri C6 flow cytometer (BD, Shanghai, China).

### MTT assay

The cells treated without or with the indicated concentration of chemical reagents were removed from incubator into laminar flow hood. 100 μl of MTT solution was added to 96 well plates. After incubating at 37°C for 3 h, the plates were centrifuged and replaced with MTT solvents (4 mM HCl, 0.1% NP40 in isopropanol). After 15 min, the optical density was read at 590 nm.

### Intracerebroventricular injection

Aβ oligomers or vehicle was injected intracerebroventricularly (i.c.v.) into C57BL/6 mice. In selected experiments, the mice were intranasal administered with ASA solution or ASA-CS before injecting (i.c.v.) Aβo. In brief, stereotaxic apparatus was adjusted to the appropriate coordinate according to the location of bregma (mediolateral, 21.0 mm; anteroposterior, 20.22 mm, and dorsoventral, 22.8 mm). The chemical reagents were slowly injected to the ventricles of mice and the injector was slowly taken out. After injection, the mice were put on the heated pad before sobering up.

### Aβ preparation

Freeze-dried Aβ monomer was dissolved in 100% HFIP to prepare 1.0 μg/μl solution. The solution was equally distributed into Eppendorf tubes and vacuum dried to store in −80°C refrigerator. The vacuum dried Aβ monomer was reconstituted with dimethyl sulfoxide (DMSO) in ultrasound water bath for 10 min to prepare 20 μg/μl solutions. Until Aβ was thoroughly dissolved, F-12 medium without phenol red was added to adjust the final concentration to 0.2 μg/μl. The solution was incubated at 4°C for 24 h before obtaining Aβo. The quality of oligomers product was controlled by Western blot using Aβ antibody (Cell Signaling Technology, Shanghai, China).

### Aβ uptake and degradation

D1A cells were incubated with Aβ for 2 h. Then, the cells were lysed to measure the uptake of Aβ by enzyme-linked immunosorbent assay (ELISA), washed with fresh medium two times and incubated in medium for an additional 46 h before determining the degradation of Aβ by ELISA.

### Tissue embedding and Immunohistochemistry

Mouse brains were collected from WT or APP/PS1 Tg mice, which is treated without or with ASA. The brains were immobilized in 4% paraformaldehyde for 48 h, soaked in 70% ethanol overnight, and soaked in 80% ethanol for 1 h at room temperature. For dehydration, the tissues were soaked sequentially in 90, 95, and 100% ethanol (twice) for 0.5 h. The tissues were then placed in xylene for 2 min−2 h and a xylene: soft wax mixture for 1.5 h and hard wax for 1.5 h. After tissue embedding, serial sections (5 μm thick) were cut using a paraffin slice (Leica, RM2235, Germany), and the sections were used for morphological determination. In detail, the slides were rehydrated with xylene and gradient ethanol, which were then eliminate endogenous peroxidase antigen for 30 min and repair the antigens for 20 min. After blocking with goat serum for 0.5 h, the slides were incubated with specific antibody overnight at 4°C. After rinsing with 0.01 M PBS for three times, the slide was incubated with secondary antibody for 2 h and streptomycin anti-biotin peroxidase for 1 h. After rinsing with PBS, the slides were visualized with DAB. In selected experiments, the nuclei of the cells in the brains were stained with hematoxylin for 1 min. The slides were finally dehydrated with gradient ethanol and cleared with xylene, which were then mounted with neutral resin before observing under microscopy.

### Plasmid construction and transfection

The siRNA targeted CDK4, p21, or TFEB was designed by siRNA selection tool (Thermo Fisher Scientific, Shanghai, China) and synthesized by GENEWIZ (Suzhou, Jiangsu, China). The genetic fragments were inserted into the lentiviral pLKO.1 vectors. After sequencing, the shRNA plasmids were purified and co-transfected with packaging vectors (psPAX2 and pMD2.G) into HEK293T cells. After 48 and 72 h, the lentiviral particles in the supernatant were concentrated through ultracentrifugation and resuspended in phosphate buffered saline (PBS) (−). For knocking down the expression of corresponding genes, the lentiviral particles that contained shRNA or control shRNA were adjusted to 10^6^–10^7^ titers prior to infecting N2a or D1A cells. For overexpression, the coding sequences of CDK4 or p21 were synthesized and inserted into the pcDNA3.1 plasmids. The vector or plasmids were transfected to N2a or D1A cells with lipofectamine 2000 according to the manufacturer’s instructions (Invitrogen, Shanghai, China).

### Nest construction

The mice were housed in cages with corncob bedding for 1 week before the nest construction test. 2 h before the onset of the dark phase of the light cycle, eight pieces of paper (5 cm × 5 cm) were introduced into the home cage to create conditions for nesting. The nests were recorded on the following mornings according to a 4-point system: (1) no biting/tearing, with random dispersion of the paper; (2) no biting/tearing of paper, with gathering in a corner/side of the cage; (3) moderate biting/tearing of paper, with gathering in a corner/side of the cage; and (4) extensive biting/tearing of paper, with gathering in a corner/side of the cage.

### Morris maze test

The experimental training phase was carried out three times per day for 10 consecutive days. During first 2 day, put the mice into the pool and record the time required for the mice to find the visible platform. In the following 7 day of training, the time was recorded for the mice to find the underwater hidden platform from the water entry point facing the pool wall. After the mice find the platform, let the mice stand on the platform for 10 s. If the mice failed to find the platform within 60 s, gently put them on the platform for 10 s. For the last day, the platform will be removed to record the passing times of the original location of platforms.

### Animal committee

All animals were handled according to the guidelines for the care and use of medical laboratory animals (Ministry of Health, Peoples Republic of China, 1998) and the guidelines of the laboratory animal ethical standards of Northeastern University.

### Statistics

All data are presented as the means ± S.E. of at least three independent experiments. The statistical significance of the differences between the means was determined using Student’s *t*-test or one-way analysis of variance, where appropriate. If the means were significantly different, multiple pairwise comparisons were performed using Tukey’s *post hoc* test.

## Results

### Aspirin attenuates amyloid plaques pathology

**FIGURE 1:**
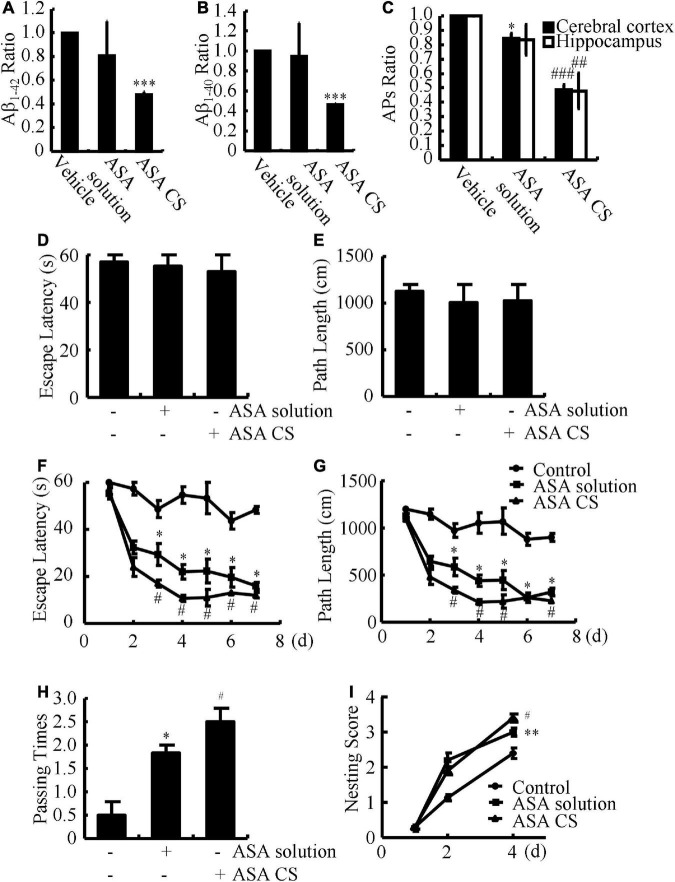
ASA-CS shows better effects on improving the cognitive decline of APP/PS1 Tg mice than that of ASA solution *via* disrupting the production and deposition of Aβ. **(A–I)** APP/PS1 Tg mice were intranasally administered with ASA solution or ASA CS (0.2 mg/kg/d) for 3 months. **(A,B)** The contents of Aβ_1–40_ and Aβ_1–42_ in the cerebral cortex and hippocampus were determined by ELISA. **(C)** The ratio of APs in the cerebral cortex and hippocampus of APP/PS1 Tg mice was determined using IHC. **(D,E)** Escape latency and path length of mice in the visible platform experiments. **(F,G)** Escape latency and path length of mice in the invisible platform experiments. **(H)** In the spatial exploration experiment, the number of times the animals crossed over the original platform was recorded by the software. **(I)** Nest construction of different group of mice were analyzed according to nesting scores. Data are presented as the means ± S.E. of independent experiments, *p < 0.05; ^**^p < 0.01; ^***^p < 0.001 compared to vehicle-treated mice, ^#^*p* < 0.05; ^##^*p* < 0.01; ^###^*p* < 0.001 compared to ASA solution-treated APP/PS1 Tg mice.

Given the potential roles of ASA in AD, we further determined the effects of ASA on the production and deposition of Aβ in APP/PS1 Tg mice. Although ASA solution could lower the average production of Aβ_1–42_ and Aβ_1–40_, ASA-CS significantly suppressed the production of Aβ_1–42_ and Aβ_1–40_ in the brains of APP/PS1 Tg mice (Figures 1A,B). To further explore the roles of ASA in the formation of APs, IHC experiments were carried out. The results showed that both ASA solution and ASA-CS inhibited the deposition of Aβ in APs (Figure 1C). Notably, ASA-CS showed relatively higher efficacy in suppressing the formation of APs than ASA solution in APP/PS1 Tg mice (Figure 1C).

### Acetylsalicylic acid-chitosan-encapsulated drug delivery system shows better effects on improving the cognitive decline of APP/PS1 Tg mice than acetylsalicylic acid solution

Based on the observation that ASA treatment attenuates Aβ aggregation and deposition of Aβ, we next investigated the relationship between ASA and memory deficits in APP/PS1 Tg mice. After 3 months of ASA treatment, we assessed spatial learning and memory abilities by the Morris maze test. In visible platform experiments, the distinct groups of mice did not show much difference, suggesting that neither ASA solution nor ASA-CS administration affected the motility and vision of mice (Figures 1D,E). In the following invisible platform experiments, ASA-CS showed better therapeutic effects on memory loss than ASA solution (Figures 1F,G). After removing the platform, ASA-CS increased the passing times of the original location of the platform compared to that of the ASA solution (Figure 1H). Moreover, nest construction is a natural inborn ability, and it became progressively impaired in APP/PS1 Tg mice; however, this impairment was reversed by ASA treatment, especially ASA-CS treatment (Figure 1I).

### Identification of differential expression of cell cycle-regulated genes in *in vitro* and *in vivo* Alzheimer’s disease models

**FIGURE 2:**
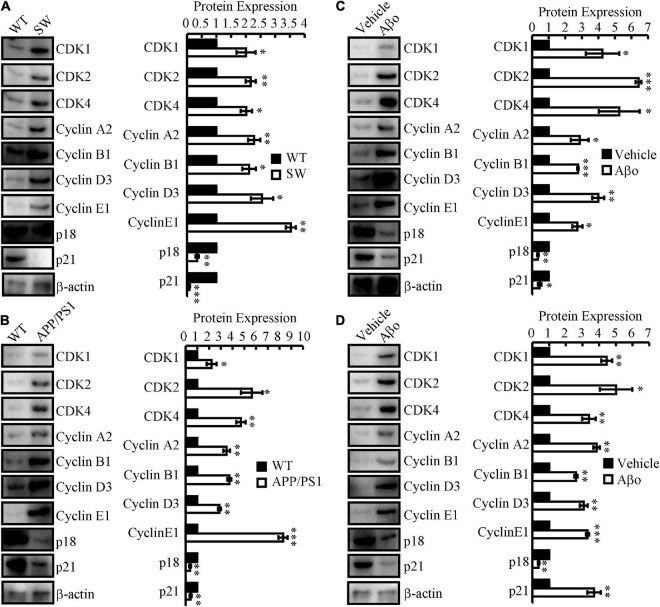
The expression of cell cycle-regulated genes in *in vitro* and *in vivo* AD models. **(A)** N2a^SW^ cells were established by transfecting N2a cells with swedish mutated APP plasmids. **(B)** The brains of 3-month-old APP/PS1 Tg mice were collected for the following experiments. **(C)** Aβo was prepared to treat N2a cells at the concentration of 20 nM. **(D)** Aβo (2 ng/5μl) was injected (i.c.v.) to the ventricles of C57BL/6 mice. **(A–D)** The total protein was extracted from the cells and brains. Western blotting was employed to detect the expression of CDK1/2/4, cyclin A2/B1/D3/E1, p18 and p21. β-actin served as an internal control. The optical density of the bands was analyzed using ImageJ software. Data are presented as the means ± S.E. of independent experiments, *p < 0.05; ^**^p < 0.01, and ^***^p < 0.001 compared to the controls.

To study differential gene expression in AD models, we initially assessed the mRNA and protein expression of cell cycle-related genes in N2a*^SW^* cells compared to that of vector-transfected cells. The results demonstrated that the mRNA and protein expression of CDK1/2/4 and CyA2/B1/D3/E1 was upregulated, whereas p18 and p21 were downregulated in N2a*^SW^* cells (Figure 2A and Table 2). In the hippocampus of 3-month-old APP/PS1 Tg mice, cell cycle-related genes were regulated similarly as those in N2a*^SW^* cells (Figure 2B and Table 3). However, the mRNA expression of cell cycle-related genes was not always consistent with those of N2a*^SW^* cells in the cerebral cortex of 3-month-old APP/PS1 Tg mice (Table 3). Given the critical roles of Aβ in AD, we further treated N2a cells and C57BL/6 mice with Aβ oligomers (Aβo). By measuring the expression of cell cycle-related genes, Aβo showed positive effects on concurrently upregulating the mRNA and protein expression of CDK1/2/4 and CyA2/B1/D3/E1 and downregulating the expression of p18 and p21 in N2a cells and the hippocampus of C57BL/6 mice (Figures 2C,D and Tables 4, 5). For their important roles in the progression of G0/G1 phase, our results suggested the re-entry of the cell cycle during the course of AD development and progression.

**TABLE 2:** The expression of cell cycle-associated genes in N2a^sw^ cells.

Genes cells	CDK1	CDK2	CDK4	CyA2	CyB1	CyD3	CyE1	p18	p21
N2a^sw^	2.10	3.21	1.24	1.65	2.06	1.32	1.66	0.34	0.86

**TABLE 3:** The expression of cell cycle-associated genes in the brains of 3-month-old APP/PS1 mice.

Genes ER	CDK1	CDK2	CDK4	CyA2	CyB1	CyD3	CyE1	p18	p21
Hippocampus	3.38	2.09	4.68	3.63	5.48	2.40	2.23	0.27	0.43
Cortex	0.44	0.71	0.82	0.08	0.09	0.54	1.93	1.07	0.44

ER, encephalic region.

**TABLE 4:** The expression of cell cycle-associated genes in Aβo-treated N2a cells.

Genes N2a cells	CDK1	CDK2	CDK4	CyA2	CyB1	CyD3	CyE1	p18	p21
Aβo	1.28	3.31	1.26	1.42	3.28	12.77	2.55	0.06	0.63

**TABLE 5:** The expression of cell cycle-associated genes in Aβo-injected (i.c.v) mice.

Genes ER	CDK1	CDK2	CDK4	CyA2	CyB1	CyD3	CyE1	p18	p21
Hippocampus	6.66	1.37	2.01	10.41	9.85	7.54	1.05	0.01	0.02
Cortex	0.99	0.46	0.09	0.21	0.07	1.17	1.01	0.30	1.56

ER, encephalic region.

### Preparation and *in vitro* release of acetylsalicylic acid-chitosan-encapsulated drug delivery system

To perform brain-targeted drug delivery and decrease the toxicity and side effects of ASA, ASA-CS was prepared for intranasal administration. ASA-CS is in a liquid state at room temperature while CS forms a cross-linking 3D colloidal gel under physiological conditions. Therefore, ASA-CS will form gels in the nasal cavity because of the thermosensitive properties of CS. By forming the gels, CS will control the slow release of ASA to maintain a stable blood drug concentration. Taking advantage of this approach, it can reduce the times of drug administration and prolong the biological half-life of drugs. In addition, intranasal administration will avoid the first pass effect of the liver and improve the bioavailability of the drug.

### Acetylsalicylic acid-chitosan-encapsulated drug delivery system showed better effects on restoring the expression of cell cycle-regulated genes than acetylsalicylic acid solution

**FIGURE 3:**
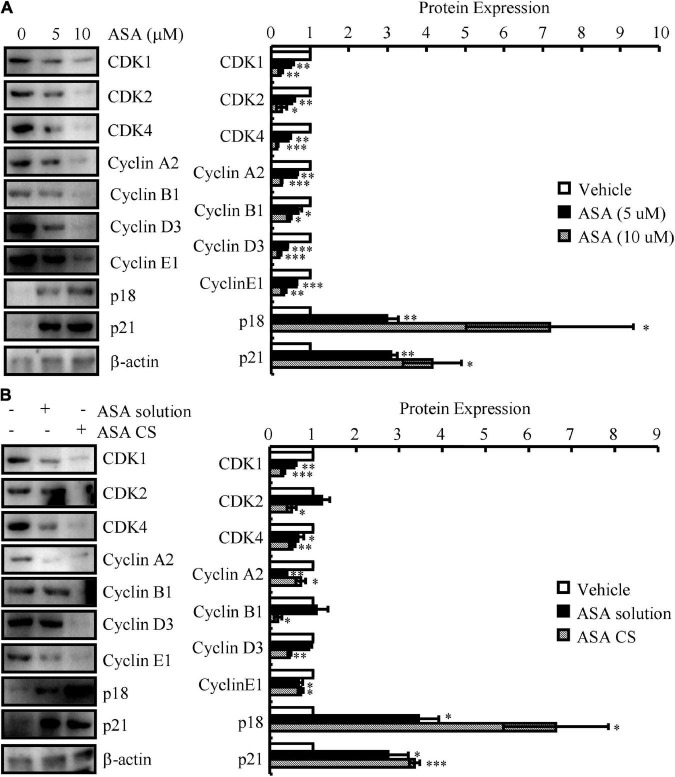
ASA-CS showed better effects on restoring the expression of cell cycle-regulated genes than that of ASA solution. **(A)** N2a cells were treated with 5 or 10 μM ASA. **(B)** ASA-CS and ASA solutions were intranasally administered to APP/PS1 Tg mice. **(A,B)** The total protein was extracted from the cells and brains. Western blotting was employed to detect the expression of CDK1/2/4, cyclin A2/B1/D3/E1, p18 and p21. β-actin served as an internal control. The optical density of the bands was analyzed using ImageJ software. Data are presented as the means ± S.E. of independent experiments, *p < 0.05; ^**^p < 0.01, and ^***^p < 0.001 compared to the controls.

We next determined the effects of ASA on the progression of the cell cycle. For this purpose, N2a cells were first treated with 5 or 10 μM ASA. Treatment with ASA decreased the mRNA and protein expression of CDK1/2/4 and CyA2/B1/D3/E1, whereas the expression of p18 and p21 was increased compared to vehicle-treated controls (Figure 3A and Table 6). To validate these *in vitro* data, ASA-CS and ASA solutions were intranasally administered to APP/PS1 Tg mice. Interestingly, ASA-CS showed better effects on restoring the expression of cell cycle-regulated genes than ASA solution (Figure 3B and Table 7). These observations suggested better therapeutic effects of ASA-CS than ASA solution on restoring the expression of cell cycle-regulated genes.

**TABLE 6:** The expression of cell cycle-associated genes in aspirin-treated N2a cells.

Genes Aspirin	CDK1	CDK2	CDK4	CyA2	CyB1	CyD3	CyE1	p18	p21
5 μM	0.46	0.87	0.61	0.66	0.96	1.16	0.91	1.13	0.95
10 μM	0.36	0.52	0.18	0.67	0.80	0.63	0.37	2.18	1.78

**TABLE 7:** The expression of cell cycle-associated genes in aspirin-administered mice.

Genes hippocampus	CDK1	CDK2	CDK4	CyA2	CyB1	CyD3	CyE1	p18	p21
Solution	0.49	0.76	0.19	0.38	0.78	0.31	0.50	2.54	1.95
Nasal gel	0.25	0.35	0.11	0.18	0.29	0.19	0.38	6.28	4.37

### Cyclin-dependent kinase 4 and p21 mediate the effects of acetylsalicylic acid on protecting neurons from cell cycle re-entry, thereby inhibiting neuronal apoptosis

**FIGURE 4:**
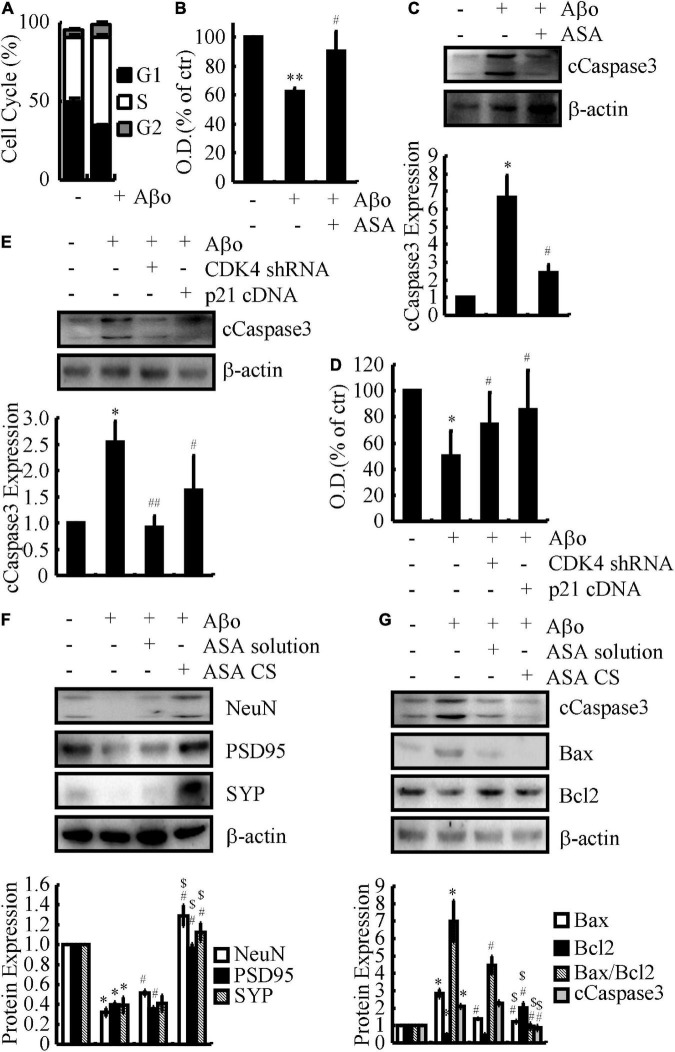
CDK4 and p21 mediate the effects of ASA on protecting neurons from cell cycle re-entry, leading to inhibit the apoptosis of neurons. **(A–C)** N2a cells were treated with Aβo (20 nM) in the absence or presence of ASA (10 μM). **(D,E)** N2a cells were treated with Aβo (20 nM) in the absence or presence of transfecting CDK4 shRNA or p21 cDNA. **(F,G)** C57BL/6 mice were injected (i.c.v.) with Aβo (2 ng/5 μl) in the absence or presence of intranasally administrating ASA solution or ASA CS. **(A)** The cell cycle was determined by flow cytometry. **(B–D)** The survival rate of N2a cells were determined by MTT assay. **(C,E–G)** The production of caspase 3 and the protein expression of NeuN, PSD95, SYP, Bax, and Bcl2 were determined by western blots. β-actin served as an internal control. The optical density of the bands was analyzed using ImageJ software. Data are presented as the means ± S.E. of independent experiments, *p < 0.05; ^**^p < 0.01 compared to vehicle-treated controls, ^#^*p* < 0.05; ^##^*p* < 0.01 compared to Aβo-treated alone, ^$^*p* < 0.05 compared to ASA solution-treated alone.

To further investigate the relationship with the cell cycle, flow cytometry experiments were carried out to determine the effects of ASA on the progression of the cell cycle in Aβo-treated N2a cells. The results demonstrated that Aβo obviously enhanced the proportion of N2a cells in S phase (Figure 4A). These observations indicate that Aβo triggers the cell cycle re-entry of neurons and suggest the consequences of cell cycle re-entry to neurons. For this purpose, N2a cells were used in the following experiments and treated with Aβo in the absence or presence of ASA. Based on a MTT assay, ASA showed beneficial effects on preventing neuronal death (Figure 4B). Mechanistically, Aβo induces the cleavage of caspase 3, which is attenuated by the addition of ASA to N2a cells (Figure 4C). Furthermore, N2a cells were transfected with either CDK4 shRNA or p21 cDNA before treatment with Aβo. Moreover, the MTT assay showed that knocking down the expression of CDK4 or ectopic expression of p21 ameliorated the effects of Aβo on inducing the death of neurons (Figure 4D). Of note, caspase 3 located downstream of CDK4 and p21 mediated the effects of Aβo on inducing neuronal death (Figure 4E).

To further validate these *in vitro* data, APP/PS1 Tg mice were administered ASA solution and ASA-CS for 3 months. Western blots assays showed that ASA-CS had better effects on restoring the protein levels of NeuN, PSD95, and SYP than ASA solution (Figure 4F). Moreover, ASA also showed the inhibitory effects of lowering the levels of caspase 3 in the brains of APP/PS1 Tg mice (Figure 4G). Along these lines, CDK4 and p21 mediate the effects of ASA on protecting neurons from cell cycle re-entry, leading to inhibition of neuronal death.

### Aspirin induces the uptake of Aβ for degradation in astrocytes

**FIGURE 5:**
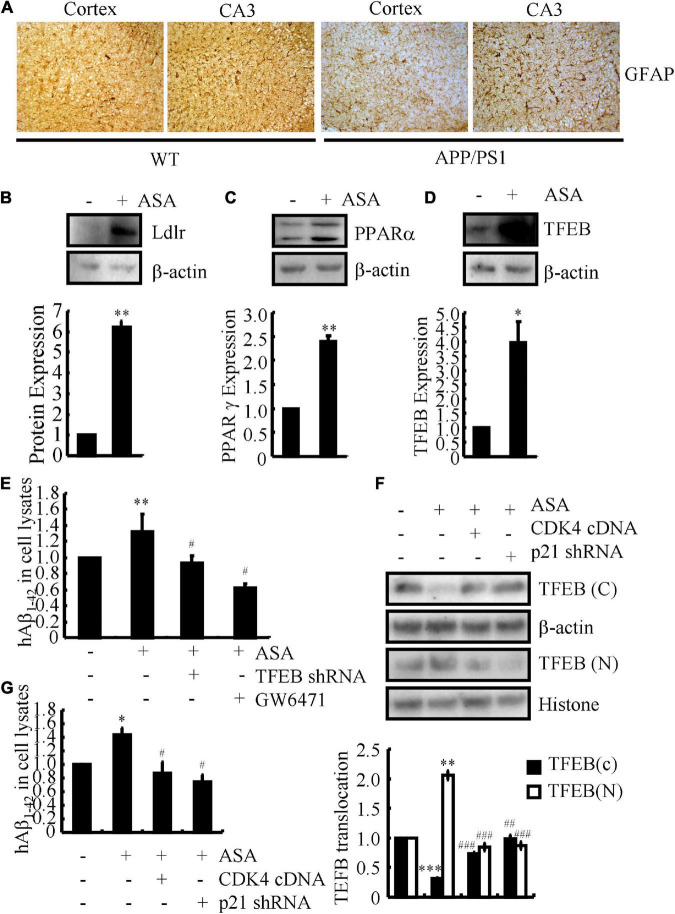
Aspirin induces the uptake of Aβ in astrocytes. **(A)** The brains of WT and APP/PS1 Tg mice were collected and sectioned after paraffin embedding. The morphology of astrocytes were determined by IHC. **(B–D)** In select experiments, D1A cells were treated with ASA (10 μM). The protein expression of Ldlr, PPARα, and TFEB were determined by western blots. β-actin served as an internal control. The optical density of the bands was analyzed using ImageJ software. **(E)** In separate experiments, D1A cells were transfected with TFEB shRNA or pre-treated with GW6471 (1 μM) before incubating in ASA (10 μM). **(F,G)** In distinct experiments, D1A cells were transfected with CDK4 cDNA and p21 shRNA before incubating with ASA (10 μM). **(E–G)** The uptake of Aβ_1–42_ was determined by ELISA. **(F)** The protein levels of TFEB in cytosol and nucleus was determined by western blots. β-actin and histone served as internal controls. The optical density of the bands was analyzed using ImageJ software. Data are presented as the means ± S.E. of independent experiments, *p < 0.05; ^**^p < 0.01; ^***^p < 0.001 compared to vehicle-treated controls, ^#^*p* < 0.05; ^##^*p* < 0.01; ^###^*p* < 0.001; compared to ASA-treated alone.

Based on the above observations, we continued to investigate the effects of ASA on the uptake of Aβ for degradation in astrocytes since astrocytes are activated in the brains of APP/PS1 Tg mice (Figure 5A). For this purpose, D1A cells were used in the following experiments. Treatment with ASA showed that the expression of Ldlr was up-regulated in D1A cells (Figure 5B), whose expression is critical for the uptake of Aβ in astrocytes. A report showed that the activation of the nuclear receptor PPARα by its agonist fenofibrate induces the expression of Ldlr; thus, we determined the effects of ASA on the activity of PPARα in astrocytes. As expected, ASA treatment induced the expression of not only PPARα but also its downstream target, TFEB, in astrocytes (Figures 5C,D). To further determine the uptake of Aβ by astrocytes, D1A cells were transfected with TFEB shRNA or treated with the PPARα antagonist GW6471 in the presence of ASA. By incubating with Aβ, the contents of Aβ in the cell lysates were determined by ELISA after 2 h of treatment with ASA. The results demonstrated that the contents of Aβ in cell lysates were induced by ASA treatment, which was blocked by TFEB knockdown and GW6471 treatment in D1A cells (Figure 5E), suggesting that ASA induces the uptake of Aβ *via* PPARα-dependent TFEB-activating mechanisms. Moreover, questions are easily raised regarding whether cell cycle re-entry is involved in regulating the uptake of Aβ by ASA treatment. For this purpose, we overexpressed CDK4 and knocked down the expression of p21 in ASA-treated D1A cells. Western blot assays showed that ASA induced the translocation of TFEB from the cytosol to the nucleus, which was partially blocked by CDK4 overexpression and p21 knockdown in D1A cells (Figure 5F). As a consequence, the uptake of Aβ was elevated by ASA treatment, which was attenuated by ectopically expressing CDK4 or p12 knockdown in D1A cells (Figure 5G). Therefore, our data revealed that cell cycle re-entry is involved in regulating the uptake of Aβ in an ASA-stimulated PPARα-dependent TFEB-activating mechanism.

**FIGURE 6:**
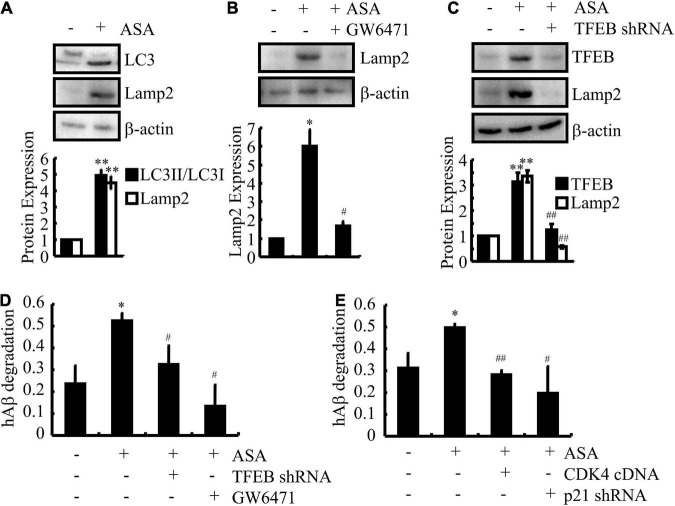
Aspirin induces the degradation of Aβ in astrocytes. **(A–D)** D1A cells were transfected with TFEB shRNA or pre-treated with GW6471 (1 μM) before incubating in ASA (10 μM). **(E)** In select experiments, D1A cells were transfected with CDK4 cDNA and p21 shRNA before incubating with ASA (10 μM). **(A–C)** The protein expression of LC3, Lamp2, and TFEB were determined by western blots. β-actin served as an internal control. The optical density of the bands was analyzed using ImageJ software. **(D,E)** The degradation of Aβ was determined by ELISA in astrocytes. Data are presented as the means ± S.E. of independent experiments, *p < 0.05; ^**^p < 0.01 compared to vehicle-treated controls, ^#^*p* < 0.05; ^##^*p* < 0.01; compared to ASA-treated alone.

Since ASA has shown its effects on inducing the uptake of Aβ in astrocytes, we investigated whether ASA has the ability to trigger the degradation of Aβ in cells. As expected, we further found that ASA treatment augmented the production of LC3II in D1A cells (Figure 6A), suggesting that autophagy might be activated by ASA to degrade Aβ. Because lysosomes are responsible for Aβ degradation, we continued to measure the activity of lysosomes in ASA-treated D1A cells. Western blot assays showed that ASA has the ability to upregulate the expression of Lamp2, a marker for lysosomes, which was blocked by GW6471 treatment or TFEB knockdown in D1A cells (Figures 6B,C). More interestingly, we found that ASA treatment for 24 h did not elevate the contents of Aβ but reduced the contents of Aβ in the cell lysates of D1A cells (Figure 6D), suggesting the ability of ASA to degrade Aβ. Moreover, GW6471 treatment or TFEB knockdown blocked the effects of ASA on inducing the degradation of Aβ in D1A cells (Figure 6D). More importantly, cell cycle re-entry was also involved in regulating the degradation of Aβ in D1A cells (Figure 6E). Collectively, these observations demonstrate that ASA attenuates AP pathology by inhibiting the production and deposition of Aβ and enhancing the uptake and degradation of Aβ in AD.

## Discussion

In the present study, we revealed an early but poorly understood mechanism for the pathogenesis of AD: neuronal cell cycle re-entry. Insights from a previous study suggested that Aβo induces microglial cell activity, which results in neuronal cell cycle re-entry *via* the TNF-α and c-Jun kinase (JNK) signaling pathways (Bhaskar et al., 2014). In particular, induction of cell cycle re-entry is toxic to terminally differentiated neurons, which is associated with neuronal cell death (TUNEL-positive) in the AD cortex (Bhaskar et al., 2014). In light of prior works, we investigated the gene regulation and cell cycle changes specifically associated with Aβo treatment and ectopic expression of APP*^SW^*, both separately and in combination and revealed changes in genes with a role in cell cycle control during the course of AD development and progression. Interestingly, ASA showed neuroprotective effects on restoring the levels of cell cycle-associated genes, which potentially contribute to the production and deposition of Aβ, leading to the cognitive decline of APP/PS1 Tg mice.

Previous studies have implied that a variety of cyclins and CDKs, such as CDK4, cyclin B1, Cdc2, and p16, are enhanced in the brains of AD patients (McShea et al., 1997; Vincent et al., 1997; Busser et al., 1998). Indeed, the expression of cell cycle proteins could be activated in neurons by a number of stimulating factors. For example, the protein level of cyclin D1 is elevated in *cis*-platinum-treated sensory neurons (Gill and Windebank, 1998). In neuronal PC12 cells, NGF deprivation stimulates the activity of Cdc2 and the expression of cyclin D1 (Gao and Zelenka, 1995), and this behavior is also observed in sympathetic neurons (Freeman et al., 1994). For AD, Aβ induces not only cell cycle re-entry but also cell death by upregulating the expression of CDK4 and the phosphorylation of Rb before entering S phase (Giovanni et al., 1999; Copani et al., 2001). In addition, Aβ_1–42_ treatment modestly upregulates the protein expression of CDK4 in SH-SY5Y cells and robustly increases the protein levels of CDK4 in tau*^P^*^301*L*^-expressing cells (Hoerndli et al., 2007). Apart from CDK4, Aβ_1–42_ also stimulates the protein expression of CDK1, CDK5, and Cdc25B and lowers the protein levels of 14-3-3, Cdc34, Chk1, cyclin D1, and Rb in SH-SY5Y cells (Hoerndli et al., 2007), which potentially contributes to the cell cycle re-entry of neurons during the course of AD development and progression.

However, the cell cycle re-entry in these neurons showed a highly unorganized nature since the profile of cyclin-CDK activity in the phase of each cell cycle is usually lost in normal dividing cells. For instance, CDK4 and p16 are expressed concurrently in these neurons and are not observed in normal dividing cells (McShea et al., 1997). Moreover, most of these cell cycle elements are expressed in the cytosol rather than in the nucleus, where they should be (Vincent et al., 1997; Ogawa et al., 2003). Although the consequence of such cell cycle re-entry is unclear, all of these aberrant regulatory factors likely lead to the inadequate or failed control of the cell cycle in these neurons, which may potentially contribute to their ultimate death in AD. It is likely that the death of PC12 cells was blocked by treatment with the CDK inhibitor flavopiridol or the expression of dominant-negative CDK4/6 in the presence of Aβ (Giovanni et al., 1999). As an inhibitor of CDK, p16 protects N cells from death caused by trophic factor deprivation (Kranenburg et al., 1996). In addition, trophic factor deprivation- and DNA damage-induced sympathetic and cortical neuronal death was blocked by a pharmacological inhibitor of the cell cycle (Farinelli and Greene, 1996; Park et al., 1996, 1997b,1998b). Virus-induced expression of CDK inhibitors, including p16 and p27, and the dominant negative forms of CDK4 and CDK6 suppress neuronal loss (Park et al., 1997a,1998a). Consistent with these prior works, we also found that CDK1, CDK4, and cyclin B1 were upregulated in Aβ-treated neuronal cells (Figure 2C and Table 3). In addition, we extended prior works and found that Aβ treatment concurrently induced the expression of CDK2 and cyclin A2/D3/E1 and reduced the expression of p18 and p21 in neuronal cells (Figure 2C and Table 3). Moreover, similar regulatory activities were further observed in N2a*^SW^* cells, Aβ-injected (i.c.v.) C57BL/6 mice and APP/PS1 Tg mice (Figures 2A,B,D and Tables 1, 2, 4), thus leading to cell cycle re-entry and apoptosis of neurons (Figure 4).

Notably, aberrantly regulated proteins in the cell cycle do not appear exclusively at the late stage of neuropathology but rather the earliest neuronal changes to occur in the disease (McShea et al., 1997; Nagy et al., 1997; Busser et al., 1998; Zhu et al., 2000). Cell cycle markers occur even prior to the appearance of gross cytopathological changes (Vincent et al., 1997). The proximal time of cell cycle re-entry appears in pre-AD patients with MCI, which is a prodromal stage of AD (Yang et al., 2003). From the point of view, it might be better to intervene in AD as early as the appearance of gross cytopathological changes or even cell cycle re-entry. Based on the above clues, ASA was selected for the current study for the following reasons. First, numerous cytokines, chemokines and inflammatory components, including COX-2, NO and IL-1β, are elevated in AD brains by activating microglia and astrocytes (Akiyama et al., 2000), which occurs earlier than Aβ deposition in different AD animals (Dudal et al., 2004). Second, COX-2 overexpression induces alteration of the neuronal cell cycle in APP/PS1 Tg mice, which provides a rational basis for targeting neuronal COX-2 in therapeutic research aimed at slowing the clinical progression of AD (Xiang et al., 2002). Third, NSAIDs have shown beneficial effects on decreasing the risk of AD in retrospective studies (McGeer et al., 1996). Although clinical trials of NSAIDs are unsuccessful, this may simply reflect its premorbid but not therapeutic effects. As an inhibitor of COX-2, ASA exhibited an ∼50% decreased risk of AD (Zandi et al., 2002). On the basis of these clues, we also made the novel discovery that ASA has the ability to protect neurons from cell cycle re-entry, which results in the apoptosis of neurons (Figure 4).

Apart from its powerful anti-inflammatory effects, ASA disrupts the oligomerization of Aβ in an *in vitro* study (Parmer et al., 2017). In light of these prior works, our data further revealed that ASA treatment clearly inhibited the production and deposition of Aβ in the brains of APP/PS1 Tg mice (Figures 1A–C). However, we could still not fill the gaps between cell cycle re-entry and Aβ deposition. This problem might be resolved by the expected experiments to knock out or overexpress CDKs, cyclins or p18/p21 in APP/PS1 Tg mice. Despite lacking connections, CDK5 has shown negative regulatory effects on the production of Aβ in HEK293 _751_APP_*SW*_ cells (Ryder et al., 2003), which is caused by the activity of BACE1 (Sadleir and Vassar, 2012). Although the roles of cell cycle proteins in the production and deposition of Aβ are quite limited, accumulating evidence has shown that Aβ is able to regulate the progression of the cell cycle and the expression of associated regulatory proteins (Wang et al., 2012). Therefore, crosstalk might exist between cell cycle re-entry and the production and deposition of Aβ during the course of AD development and progression.

In addition to the above functions, ASA also shows the ability to enhance astrocyte uptake of Aβ, leading to the degradation of Aβ in lysosomes (Figure 5). In line with our observations, oral administration of ASA can upregulate lysosomal markers, including Lamp2, in the hippocampus of 5 × fAD mice, which decreases the loading of APs (Chandra et al., 2018). During this process, TFEB was found to be transcriptionally upregulated by a PPARα-dependent mechanism under the ASA treatment (Figures 5C,D). Consistent with our findings, TFEB activation enhances the function of lysosomes in degrading APP, which results in decreased production of Aβ and formation of APs (Xiao et al., 2015). On the other hand, TFEB can attenuate the pathogenesis of AD by facilitating the uptake and lysosomal degradation of Aβ in astrocytes (Xiao et al., 2014), which is consistent with our observations in ASA-treated astrocytes (Figure 6D). In addition, Ldlr was elevated by ASA treatment in astrocytes (Figure 5B). Supporting our results, Ldlr has been implicated in the direct binding and internalization of Aβ by astrocytes (Basak et al., 2012), whose deficiency reduces the responses of glial cells and increases AP burden in 5 × fAD mice (Katsouri and Georgopoulos, 2011). In addition, PPARα activation by its agonist fenofibrate is also involved in Ldlr expression (Huang et al., 2008). More interestingly, CDK4 regulated the uptake and degradation of Aβ by regulating TFEB in astrocytes (Figures 5G, 6E). Of note, these observations were further supported by a previous study, suggesting that CDK4 interacts with phosphorylated TFEB, which inactivates them by promoting their shuttling to the cytoplasm (Yin et al., 2010).

Given the beneficial effects of ASA on AD, we further found that ASA has the ability to improve the cognitive decline of APP/PS1 Tg mice (Figures 1D–I). In line with our observations, ASA enhanced memory in an AlCl_3_-induced mouse model of neurotoxicity (Rizwan et al., 2016). In addition, ASA has been reported to reduce the activity of NF-κB *via* acetylation of COX-2, which results in enhanced phagocytosis of microglial cells to facilitate the clearance of Aβ and cognitive improvement in Tg2576 mice (Medeiros et al., 2013). Moreover, high-dose ASA is effective in lowering the prevalence of AD and improving cognition (Nilsson et al., 2003). Therefore, ASA showed protective effects against AD by inhibiting the cell cycle re-entry of neurons.

## Data availability statement

The original contributions presented in this study are included in the article/Supplementary material, further inquiries can be directed to the corresponding author.

## Ethics statement

All animals were handled according to the guidelines for the care and use of medical laboratory animals (Ministry of Health, Peoples Republic of China, 1998) and the guidelines of the laboratory animal ethical standards of Northeastern University.

## Author contributions

P-PG and W-YD conceived and performed all of the experiments, participated in the design of the study, and wrote the manuscript. PW conceived the experiments, interpreted the data, and wrote the manuscript of the study. All authors contributed to the article and approved the submitted version.

## References

[B1] Akiyama H. Arai T. Kondo H. Tanno E. Haga C. Ikeda K. (2000). Cell mediators of inflammation in the Alzheimer disease brain. *Alzheimer Dis. Assoc. Disord.* 14(Suppl. 1) S47–S53. 10.1097/00002093-200000001-00008 10850730

[B2] Asanuma M. Nishibayashi-Asanuma S. Miyazaki I. Kohno M. Ogawa N. (2001). Neuroprotective effects of non-steroidal anti-inflammatory drugs by direct scavenging of nitric oxide radicals. *J. Neurochem.* 76 1895–1904. 10.1046/j.1471-4159.2001.00205.x 11259508

[B3] Basak J. M. Verghese P. B. Yoon H. Kim J. Holtzman D. M. (2012). Low-density lipoprotein receptor represents an apolipoprotein E-independent pathway of Abeta uptake and degradation by astrocytes. *J. Biol. Chem.* 287 13959–13971. 10.1074/jbc.M111.288746 22383525 PMC3340151

[B4] Bhaskar K. Maphis N. Xu G. Varvel N. H. Kokiko-Cochran O. N. Weick J. P. (2014). Microglial derived tumor necrosis factor-alpha drives Alzheimer’s disease-related neuronal cell cycle events. *Neurobiol. Dis.* 62 273–285. 10.1016/j.nbd.2013.10.007 24141019 PMC3877710

[B5] Bonda D. J. Evans T. A. Santocanale C. Llosa J. C. Vina J. Bajic V. P. (2009). Evidence for the progression through S-phase in the ectopic cell cycle re-entry of neurons in Alzheimer disease. *Aging* 1 382–388. 10.18632/aging.100044 19946466 PMC2783633

[B6] Busser J. Geldmacher D. S. Herrup K. (1998). Ectopic cell cycle proteins predict the sites of neuronal cell death in Alzheimer’s disease brain. *J. Neurosci.* 18 2801–2807. 10.1523/JNEUROSCI.18-08-02801.1998 9525997 PMC6792587

[B7] Chandra S. Jana M. Pahan K. (2018). Aspirin induces lysosomal biogenesis and attenuates amyloid plaque pathology in a mouse model of Alzheimer’s disease *via* PPARalpha. *J. Neurosci.* 38 6682–6699. 10.1523/JNEUROSCI.0054-18.2018 29967008 PMC6067079

[B8] Chen Y. X. Zhang X. R. Xie W. F. Li S. (2004). Effects of taurine on proliferation and apoptosis of hepatic stellate cells *in vitro*. *Hepatobiliary Pancreat. Dis. Int.* 3 106–109. 14969850

[B9] Copani A. Uberti D. Sortino M. A. Bruno V. Nicoletti F. Memo M. (2001). Activation of cell-cycle-associated proteins in neuronal death: A mandatory or dispensable path? *Trends Neurosci.* 24 25–31. 10.1016/S0166-2236(00)01663-5 11163884

[B10] Dudal S. Krzywkowski P. Paquette J. Morissette C. Lacombe D. Tremblay P. (2004). Inflammation occurs early during the Abeta deposition process in TgCRND8 mice. *Neurobiol. Aging* 25 861–871. 10.1016/j.neurobiolaging.2003.08.008 15212840

[B11] Evans T. A. (2007). BRCA1 may modulate neuronal cell cycle re-entry in Alzheimer disease. *Int. J. Med. Sci.* 4 140–145. 10.7150/ijms.4.140 17505559 PMC1868658

[B12] Eve R.-G. Leroux J. C. (2004). In situ-forming hydrogels–review of temperature-sensitive systems. *Eur. J. Pharm. Biopharm.* 58 409–426. 10.1016/j.ejpb.2004.03.019 15296964

[B13] Farinelli S. E. Greene L. A. (1996). Cell cycle blockers mimosine, ciclopirox, and deferoxamine prevent the death of PC12 cells and postmitotic sympathetic neurons after removal of trophic support. *J. Neurosci.* 16 1150–1162. 10.1523/JNEUROSCI.16-03-01150.1996 8558244 PMC6578784

[B14] Freeman R. S. Estus S. Johnson E. M. Jr. (1994). Analysis of cell cycle-related gene expression in postmitotic neurons: Selective induction of Cyclin D1 during programmed cell death. *Neuron* 12 343–355. 10.1016/0896-6273(94)90276-3 8110463

[B15] Gao C. Y. Zelenka P. S. (1995). Induction of cyclin B and H1 kinase activity in apoptotic PC12 cells. *Exp. Cell Res.* 219 612–618. 10.1006/excr.1995.1271 7641812

[B16] Ghosh A. Jana M. Modi K. Gonzalez F. J. Sims K. B. Berry-Kravis E. (2015). Activation of peroxisome proliferator-activated receptor alpha induces lysosomal biogenesis in brain cells: Implications for lysosomal storage disorders. *J. Biol. Chem.* 290 10309–10324. 10.1074/jbc.M114.610659 25750174 PMC4400343

[B17] Gill J. S. Windebank A. J. (1998). Cisplatin-induced apoptosis in rat dorsal root ganglion neurons is associated with attempted entry into the cell cycle. *J. Clin. Invest.* 101 2842–2850. 10.1172/JCI1130 9637718 PMC508875

[B18] Giovanni A. Wirtz-Brugger F. Keramaris E. Slack R. Park D. S. (1999). Involvement of cell cycle elements, cyclin-dependent kinases, pRb, and E2F x DP, in B-amyloid-induced neuronal death. *J. Biol. Chem.* 274 19011–19016. 10.1074/jbc.274.27.19011 10383401

[B19] Hoerndli F. J. Pelech S. Papassotiropoulos A. Gotz J. (2007). Abeta treatment and P301L tau expression in an Alzheimer’s disease tissue culture model act synergistically to promote aberrant cell cycle re-entry. *Eur. J. Neurosci.* 26 60–72. 10.1111/j.1460-9568.2007.05618.x 17587323

[B20] Huang Z. Zhou X. Nicholson A. C. Gotto A. M. Jr. Hajjar D. P. Han J. (2008). Activation of peroxisome proliferator-activated receptor-alpha in mice induces expression of the hepatic low-density lipoprotein receptor. *Br. J. Pharmacol.* 155 596–605. 10.1038/bjp.2008.331 18852694 PMC2518458

[B21] Katsouri L. Georgopoulos S. (2011). Lack of LDL receptor enhances amyloid deposition and decreases glial response in an Alzheimer’s disease mouse model. *PLoS One* 6:e21880. 10.1371/journal.pone.0021880 21755005 PMC3130747

[B22] Khurana V. Feany M. B. (2007). Connecting cell-cycle activation to neurodegeneration in Drosophila. *Biochim. Biophys. Acta* 1772 446–456. 10.1016/j.bbadis.2006.10.007 17141486 PMC2562667

[B23] Kranenburg O. Van Der Eb A. J. Zantema A. (1996). Cyclin D1 is an essential mediator of apoptotic neuronal cell death. *EMBO J.* 15 46–54. 10.1002/j.1460-2075.1996.tb00332.x 8598205 PMC449916

[B24] Laferla F. M. Oddo S. (2005). Alzheimer’s disease: Aβ, tau and synaptic dysfunction. *Trends Mol. Med.* 11 170–176. 10.1016/j.molmed.2005.02.009 15823755

[B25] Lamkin T. J. Chin V. Yen A. (2006). All-trans retinoic acid induces p62DOK1 and p56DOK2 expression which enhances induced differentiation and G0 arrest of HL-60 leukemia cells. *Am. J. Hematol.* 81 603–615. 10.1002/ajh.20667 16823827

[B26] Legler D. F. Bruckner M. Uetz-Von Allmen E. Krause P. (2010). Prostaglandin E2 at new glance: Novel insights in functional diversity offer therapeutic chances. *Int. J. Biochem. Cell Biol.* 42 198–201. 10.1016/j.biocel.2009.09.015 19788928

[B27] Liu D. X. Greene L. A. (2001). Regulation of neuronal survival and death by E2F-dependent gene repression and derepression. *Neuron* 32 425–438. 10.1016/S0896-6273(01)00495-0 11709154

[B28] Love S. (2003). Neuronal expression of cell cycle-related proteins after brain ischaemia in man. *Neurosci. Lett.* 353 29–32. 10.1016/j.neulet.2003.09.004 14642430

[B29] McGeer P. L. Schulzer M. Mcgeer E. G. (1996). Arthritis and anti-inflammatory agents as possible protective factors for Alzheimer’s disease: A review of 17 epidemiologic studies. *Neurology* 47 425–432. 10.1212/WNL.47.2.425 8757015

[B30] McShea A. Harris P. L. Webster K. R. Wahl A. F. Smith M. A. (1997). Abnormal expression of the cell cycle regulators P16 and CDK4 in Alzheimer’s disease. *Am. J. Pathol.* 150 1933–1939. 9176387 PMC1858317

[B31] McShea A. Lee H. G. Petersen R. B. Casadesus G. Vincent I. Linford N. J. (2007). Neuronal cell cycle re-entry mediates Alzheimer disease-type changes. *BBA Mol. Basis Dis.* 1772 467–472. 10.1016/j.bbadis.2006.09.010 17095196

[B32] Medeiros R. Kitazawa M. Passos G. F. Baglietto-Vargas D. Cheng D. Cribbs D. H. (2013). Aspirin-triggered lipoxin A4 stimulates alternative activation of microglia and reduces Alzheimer disease-like pathology in mice. *Am. J. Pathol.* 182 1780–1789. 10.1016/j.ajpath.2013.01.051 23506847 PMC3644736

[B33] Morinaga Y. Hayashi H. Takeuchi A. Onozaki K. (1990). Antiproliferative effect of interleukin 1 (IL-1) on tumor cells: G0-G1 arrest of a human melanoma cell line by IL-1. *Biochem. Biophys. Res. Commun.* 173 186–192. 10.1016/S0006-291X(05)81039-3 2256913

[B34] Murakami M. Goto T. Saito Y. Goto S. Kochi M. Ushio Y. (2001). The inhibitory effect of simvastatin on growth in malignant gliomas–with special reference to its local application with fibrin glue spray *in vivo*. *Int. J. Oncol.* 19 525–531. 10.3892/ijo.19.3.525 11494031

[B35] Nagy Z. Esiri M. M. Smith A. D. (1997). Expression of cell division markers in the hippocampus in Alzheimer’s disease and other neurodegenerative conditions. *Acta Neuropathol.* 93 294–300. 10.1007/s004010050617 9083562

[B36] Nilsson S. E. Johansson B. Takkinen S. Berg S. Zarit S. Mcclearn G. (2003). Does aspirin protect against Alzheimer’s dementia? A study in a Swedish population-based sample aged > or = 80 years. *Eur. J. Clin. Pharmacol.* 59 313–319. 10.1007/s00228-003-0618-y 12827329

[B37] Ogawa O. Lee H. G. Zhu X. Raina A. Harris P. L. Castellani R. J. (2003). Increased p27, an essential component of cell cycle control, in Alzheimer’s disease. *Aging Cell* 2 105–110. 10.1046/j.1474-9728.2003.00042.x 12882323

[B38] Park D. S. Farinelli S. E. Greene L. A. (1996). Inhibitors of cyclin-dependent kinases promote survival of post-mitotic neuronally differentiated PC12 cells and sympathetic neurons. *J. Biol. Chem.* 271 8161–8169. 10.1074/jbc.271.14.8161 8626506

[B39] Park D. S. Morris E. J. Greene L. A. Geller H. M. (1997b). G1/S cell cycle blockers and inhibitors of cyclin-dependent kinases suppress camptothecin-induced neuronal apoptosis. *J. Neurosci.* 17 1256–1270. 10.1523/JNEUROSCI.17-04-01256.1997 9006970 PMC6793728

[B40] Park D. S. Levine B. Ferrari G. Greene L. A. (1997a). Cyclin dependent kinase inhibitors and dominant negative cyclin dependent kinase 4 and 6 promote survival of NGF-deprived sympathetic neurons. *J. Neurosci.* 17 8975–8983. 10.1523/JNEUROSCI.17-23-08975.1997 9364045 PMC6573623

[B41] Park D. S. Morris E. J. Stefanis L. Troy C. M. Shelanski M. L. Geller H. M. (1998b). Multiple pathways of neuronal death induced by DNA-damaging agents, NGF deprivation, and oxidative stress. *J. Neurosci.* 18 830–840. 10.1523/JNEUROSCI.18-03-00830.1998 9437005 PMC6792759

[B42] Park D. S. Morris E. J. Padmanabhan J. Shelanski M. L. Geller H. M. Greene L. A. (1998a). Cyclin-dependent kinases participate in death of neurons evoked by DNA-damaging agents. *J. Cell Biol.* 143 457–467. 10.1083/jcb.143.2.457 9786955 PMC2132832

[B43] Parmer M. Milan S. Torabi A. (2017). Calcitonin-negative neuroendocrine tumor of the thyroid. *Int. J. Surg. Pathol.* 25 191–194. 10.1177/1066896916670989 27658647

[B44] Pomponi M. Di Gioia A. Bria P. Pomponi M. F. (2008). Fatty aspirin: A new perspective in the prevention of dementia of Alzheimer’s type? *Curr. Alzheimer Res.* 5 422–431. 10.2174/156720508785908892 18855583

[B45] Rizwan S. Idrees A. Ashraf M. Ahmed T. (2016). Memory-enhancing effect of aspirin is mediated through opioid system modulation in an AlCl3-induced neurotoxicity mouse model. *Exp. Ther. Med.* 11 1961–1970. 10.3892/etm.2016.3147 27168835 PMC4840773

[B46] Ryder J. Su Y. Liu F. Li B. Zhou Y. Ni B. (2003). Divergent roles of GSK3 and CDK5 in APP processing. *Biochem. Biophys. Res. Commun.* 312 922–929. 10.1016/j.bbrc.2003.11.014 14651959

[B47] Sadleir K. R. Vassar R. (2012). Cdk5 protein inhibition and Abeta42 increase BACE1 protein level in primary neurons by a post-transcriptional mechanism: Implications of CDK5 as a therapeutic target for Alzheimer disease. *J. Biol. Chem.* 287 7224–7235. 10.1074/jbc.M111.333914 22223639 PMC3293556

[B48] Sangfelt O. Erickson S. Castro J. Heiden T. Gustafsson A. Einhorn S. (1999). Molecular mechanisms underlying interferon-alpha-induced G0/G1 arrest: CKI-mediated regulation of G1 Cdk-complexes and activation of pocket proteins. *Oncogene* 18 2798–2810. 10.1038/sj.onc.1202609 10362250

[B49] Snape M. Lee H. G. Casadesus G. Smith M. A. (2009). Cell cycle aberrations in Alzheimer’s disease: A novel therapeutic opportunity. *Expert Rev. Neurother.* 9 1579–1580. 10.1586/ern.09.113 19903017

[B50] Tortosa E. Avila J. Perez M. (2006). Acetylsalicylic acid decreases tau phosphorylation at serine 422. *Neurosci. Lett.* 396 77–80. 10.1016/j.neulet.2005.11.066 16386371

[B51] Vincent I. Jicha G. Rosado M. Dickson D. W. (1997). Aberrant expression of mitotic cdc2/cyclin B1 kinase in degenerating neurons of Alzheimer’s disease brain. *J. Neurosci.* 17 3588–3598. 10.1523/JNEUROSCI.17-10-03588.1997 9133382 PMC6573674

[B52] Wang J. Zhang Y. J. Du S. (2012). The protective effect of curcumin on Aβ induced aberrant cell cycle reentry on primary cultured rat cortical neurons. *Eur. Rev. Med. Pharmacol.* 16 445–454. 22696871

[B53] Xiang Z. Ho L. Valdellon J. Borchelt D. Kelley K. Spielman L. (2002). Cyclooxygenase (COX)-2 and cell cycle activity in a transgenic mouse model of Alzheimer’s disease neuropathology. *Neurobiol. Aging* 23 327–334. 10.1016/S0197-4580(01)00282-2 11959394

[B54] Xiao Q. Yan P. Ma X. Liu H. Perez R. Zhu A. (2014). Enhancing astrocytic lysosome biogenesis facilitates Abeta clearance and attenuates amyloid plaque pathogenesis. *J. Neurosci.* 34 9607–9620. 10.1523/JNEUROSCI.3788-13.2014 25031402 PMC4099542

[B55] Xiao Q. Yan P. Ma X. Liu H. Perez R. Zhu A. (2015). Neuronal-targeted TFEB accelerates lysosomal degradation of app, reducing abeta generation and amyloid plaque pathogenesis. *J. Neurosci.* 35 12137–12151. 10.1523/JNEUROSCI.0705-15.2015 26338325 PMC4556784

[B56] Yang Y. Mufson E. J. Herrup K. (2003). Neuronal cell death is preceded by cell cycle events at all stages of Alzheimer’s disease. *J. Neurosci.* 23 2557–2563. 10.1523/JNEUROSCI.23-07-02557.2003 12684440 PMC6742098

[B57] Yin Q. Jian Y. Xu M. Huang X. Yang C. (2010). CDK4/6 regulate lysosome biogenesis through TFEB/TFE3. *J. Cell Biol.* 219:e201911036. 10.1083/jcb.201911036 32662822 PMC7401801

[B58] Zandi P. P. Anthony J. C. Hayden K. M. Mehta K. Mayer L. Breitner J. C. (2002). Reduced incidence of AD with NSAID but not H2 receptor antagonists: The Cache County Study. *Neurology* 59 880–886. 10.1212/WNL.59.6.880 12297571

[B59] Zhang Y. D. Zhao J. J. (2015). TFEB participates in the Abeta-induced pathogenesis of Alzheimer’s disease by regulating the autophagy-lysosome pathway. *DNA Cell Biol.* 34 661–668. 10.1089/dna.2014.2738 26368054

[B60] Zhu X. Rottkamp C. A. Raina A. K. Brewer G. J. Ghanbari H. A. Boux H. (2000). Neuronal CDK7 in hippocampus is related to aging and Alzheimer disease. *Neurobiol. Aging* 21 807–813. 10.1016/S0197-4580(00)00217-7 11124424

