# Supplementary material for: Molecular mechanism of acetylsalicylic acid in improving learning and memory impairment in APP/PS1 transgenic mice by inhibiting the abnormal cell cycle re-entry of neurons
Source: Front Mol Neurosci. 2022 Oct 3;15:1006216. doi: 10.3389/fnmol.2022.1006216 (PMC9575964; doi:10.3389/fnmol.2022.1006216)
Supplement: Supplementary file 1 [file Table_1.docx]

**Supplemental Table 1.** The expression of Cyclin B2/B3 and p27 in different *in vitro* and *in vivo* AD models.

| **Models**  **Genes** | **N2a^sw^** | **APP/PS1^Hippo^** | **APP/PS1^Cortex^** | **N2a^Aβo^** | **C57BL/6^Aβo^ Hippo** | **C57BL/6^Aβo^ Cortex** |
| --- | --- | --- | --- | --- | --- | --- |
| Cyclin B2 | 0.62 | 2.62 | 0.46 | 0.53 | 4.84 | 1.37 |
| Cyclin B3 | 0.42 | 1.10 | 1.49 | 1.37 | 1.73 | 1.31 |
| p27 | 1.39 | 2.06 | 1.39 |  |  |  |
| **Models**  **Genes** | **N2a^ASA (5 μM)^** | **N2a^ASA (10 μM)^** | **APP/PS1^ASA solu^ Hippo** | **APP/PS1^ASA solu^ Cortex** | **APP/PS1^ASA CS^ Hipp** | **APP/PS1^ASA CS^ Cortex** |
| Cyclin B2 |  |  | 6.04 | 13.50 |  |  |
| Cyclin B3 | 2.21 | 1.46 | 0.19 | 2.65 | 1.00 |  |
| p27 |  |  | 0.99 | 2.69 |  |  |
